# Pulmonary rehabilitation in Africa: where are we? a multimethod study

**DOI:** 10.11604/pamj.2022.42.78.31954

**Published:** 2022-05-27

**Authors:** Abbi-Monique Mamani Bilungula, Patrick Katoto, Rik Gosselink, Jean-Marie Ntumba Kayembe, Daniel Langer

**Affiliations:** 1 Department of Physical Medicine and Rehabilitation, Division of Cardio-Pulmonary Rehabilitation, Faculty of Medicine, University of Kinshasa, Lemba, B-190, Kinshasa, Democratic Republic of the Congo,; 2 Department Rehabilitation Sciences, Faculty Movement and Rehabilitation Sciences, Katholieke Universiteit of Leuven, University Hospitals Leuven, Leuven, Belgium,; 3 Cochrane South Africa, South African Medical Research Council, Cape Town, South Africa; 4 Department of Global Health, Faculty of Medicine and Health Sciences, Stellenbosch University, Cape Town, South Africa,; 5 Centre for Tropical Diseases and Global Health, Faculty of Medicine, Catholic University of Bukavu, Bukavu, Democratic Republic of the Congo,; 6 Department of Pneumology, Faculty of Medicine, University of Kinshasa, Lemba, Kinshasa, Democratic Republic of the Congo

**Keywords:** Exercise, physiotherapy, pulmonary rehabilitation, Africa

## Abstract

Pulmonary rehabilitation (PR) is an integral part of the management of patients with chronic respiratory diseases. However, there is limited information available on the effectiveness and practice of PR in Africa. This study was conducted to examine the prevalence, structure, and organization of PR in Africa, as well as its substance and claimed efficacy. We conducted a multimethod study involving systematic review of PR studies (obtained from PubMed, Google Scholar, and Cochrane databases) and a web-based survey of African healthcare professionals engaged in PR (using a standardized questionnaire). The review included papers on at least one component of PR in Africa and excluded those on PR from other continents or assessing pulmonary disorders in general without PR, cardio-rehabilitation, or physiotherapy practice in general in Africa. The Cochrane risk of bias and the Newcastle Ottawa scale instruments were used to assess the quality of included studies. We narratively synthesised data across the studies to produce a holistic picture. Of the 14 studies included for qualitative synthesis, seven were randomized controlled trials on the effectiveness of PR treatments with a total number of 333 participants. Of the 39 surveys mailed to health professionals working in Africa, only 14 (35.8%) were returned. We found aerobic exercise and breathing exercises were the most used technique and that quality of life, exercise capacity, and lung function improved significantly after PR treatments. There were differences in the duration, frequency, and length of the programs across the continent. Half of the respondents indicated that their institutions had one or more PR programs for inpatient, outpatient, maintenance, and/or home-based programs. Additionally, aerobic activities, upper and lower extremity strength training were the most frequently used exercise modalities in PR programs, followed by breathing exercises. Pulmonary rehabilitation is understudied in Africa, but it has been linked to improved lung function, exercise capacity, and quality of life. There is a need to invest in techniques tailored to the continent to enhance the implementation of pulmonary rehabilitation in Africa.

## Introduction

In developing countries, public health is facing the impact of chronic respiratory diseases (CRDs) which is challenging because of their frequency, severity, projected trends and economic impact [1]. In low and middle income countries, CRDs are significantly involved in morbidity and mortality [2]. Detailed information on CRDs in Africa is limited. However, the scarce data suggests that the burden in this setting may be high: The international BOLD (Burden of Obstructive Lung Disease) study demonstrated a considerably higher prevalence of moderate to severe airway obstruction among adults aged 40 years or older in Cape Town, South Africa (19.1%) compared with that seen in Western Europe and North American settings (5.9-14.3%) [3]. Moreover, a systematic review summarizing data from 13 studies conducted in Africa, showed a median prevalence of chronic obstructive pulmonary diseases (COPD) in persons? 40 years of 13.4% (IQR: 9.4%-22.1%) with no significant differences in gender or the year of the study on the reported prevalence of COPD [4]. When applied to the appropriate age group (40 years or more), which accounted for 196.4 million people in Africa in 2010, the estimated prevalence translates into 26.3 million (18.5-43.4 million) cases of COPD suggesting an increase of 31.5% over a decade that is attributable to ageing of the African population alone (if compared to year 2000) [4].

The management of COPD is multidisciplinary [5] and pulmonary rehabilitation (PR) is an evidence-based treatment for CRDs [6]. PR program includes exercise training, education, psychosocial and behavioural intervention, nutritional education, outcome assessment and promotion of long-term adherence to rehabilitation recommendations [5]. PR should be prescribed for patients with chronic respiratory conditions like dyspnoea or other respiratory symptoms, which reduced tolerance to exercise and decreased daily activities [5]. PR is an integral part of the management of patients suffering from CRDs [7], also other than COPD [6], as well as restrictive lung disease [8]. However, there is not much information on the practice of PR in Africa. Though it is evident that in some African countries, such as South Africa, there are hospitals or healthcare with well-established PR programs [9]. Thus, we aimed to ascertain the nature of current PR practice in Africa to provide an overview of its organization (structure and content) as well as of its effectiveness to inform practice and policy.

## Methods

We conducted a multimethod study combining a systematic review of the literature and a cross-sectional study among health workers involved in PR practice in Africa.

### Systematic review

**Search strategy and selection criteria:** we searched the following electronic databases: PubMed, Google scholar, Cochrane. In general, databases were searched with a combination of terms (respiratory OR pulmonary) AND (rehabilitation OR exercise OR physiotherapy) AND (Africa OR Afrique) and derived keywords, including variations of the following terms: “chronic obstructive pulmonary disease OR COPD”, “asthma”, “tuberculosis”, “physiotherapy”, “exercise”, “rehabilitation”, “Africa”, “Afrique” or North Africa or “sub-Saharan-Africa”. No language restriction was applied; the timeframe of the search included all records from the electronic database inception December 2018 to September 30, 2020, and an update was realisedend of January 2021. In addition, grey literature was searched, and individual authors or organization were contacted for data completeness. The PR definition was described as “comprehensive intervention based on thorough patient assessment followed by patient-tailored therapies that include, but are not limited to, exercise training, education, and behaviour change, designed to improve the physical and psychological condition of people with chronic respiratory disease and to promote the long-term adherence to health-enhancing behaviours” [6]. We considered that every paper reporting on a part of PR in Africa was eligible for inclusion in the review. Consequently, studies reporting data on PR from other continents than Africa, on pulmonary diseases in general and not including PR, on other therapy than PR for pulmonary diseases, on cardio-rehabilitation and on physiotherapy practice in general in Africa were excluded. Our study population included children, adults or elderly residing in Africa. We assessed lung function [forced vital capacity (FVC), forced expiratory volume in one second (FEV1), and peak expiratory flow (PEF)] in randomized trials, as well as physical activity [six-minute walking distance (6MWD)] and quality of life (QoL). These findings were analysed to examine the effectiveness of the public relations programs provided in each study. The outcomes of patients with CRDs or a disease that results in a respiratory deficit were sought because this population is the target demographic for a PR program and the treatments employed in each trial. We used Zotero to manage references and eliminate duplications. Two reviewers (AB and PK) evaluated the eligibility of studies. In cases of discrepancy a third reviewer (JK) provided arbitration. All papers were screened on the content (patient population, country, aim of the paper, findings) and summed up in a table to provide an overview of the kind of research on PR in Africa in observational studies. A second table was built to report on the design of the study, population, intervention, outcome, and key findings of effectiveness studies.

**Data analysis:** we performed a narrative synthesis and produced summary tables of findings of included papers because methodological heterogeneity in exposure and outcome assessment precluded meta-analyses. This systematic review was reported according to the Preferred Reporting Items for Systematic Reviews and Meta-analyses (PRISMA) guideline [10].

**Risk of bias:** two reviewers (AB and PK) independently assessed the papers' risk of bias and then debated the appropriate interpretation. In case of discrepancies, a third reviewer (JK) was asked to provide the final judgment. The Cochrane risk of bias instrument was used to assess the risk of bias in randomized trials studies, while the Newcastle Ottawa scale was utilized for observational studies.

### Cross-sectional study

**Study design, population, and settings:** a cross-sectional study (from December 2018 to February 2020) was conducted using the questionnaire from a Canadian survey of 2005 published by Brooks *et al*. [11]. We obtained the ethical committee approval from the National Health Ethical Committee of Democratic Republic of Congo (amended as n°258/CNES/BN/PMMF/2021). This questionnaire contains 32-items that we used to assess PR program components, healthcare professionals working on PR, outcome measures and follow-up components in Africa. No personal information was asked. Health professionals (physiotherapists, physicians, and nurses) were identified online (through Google searches by country or by publication in the aforementioned databases) or via national, regional, and international respiratory societies (South African Physiotherapy Society, Algerian Physical Medicine and Rehabilitation Society, Pan African Thoracic Society and, European Respiratory Society). They were then asked if they have a functional unit of PR and if they would volunteer for the study. The survey was distributed through email to individuals who indicated an interest in participating.

**Statistical analysis:** data were collected via Microsoft Excel spreadsheet. SPSS statistical program was used to perform statistical analysis. Descriptive statistics such as the mean (standard deviation) and median (interquartile ranges) were calculated for data summary. The distribution of PR programs was calculated by expressing the number of facilities (public hospitals and private hospitals) per city and presenting the number of rehabilitation programs available per city. All programs mentioned in the survey (inpatient, outpatient, home-based, maintenance) and online support (other program) were present in different facilities in South Africa. **Ethical approval:** we obtained the ethical approval from the National Health Ethical Committee of Democratic Republic of Congo (amendment n°258/CNES/BN/PMMF/2021).

## Current status of knowledge

### Literature review on pulmonary rehabilitation in Africa

**Figure 1:**
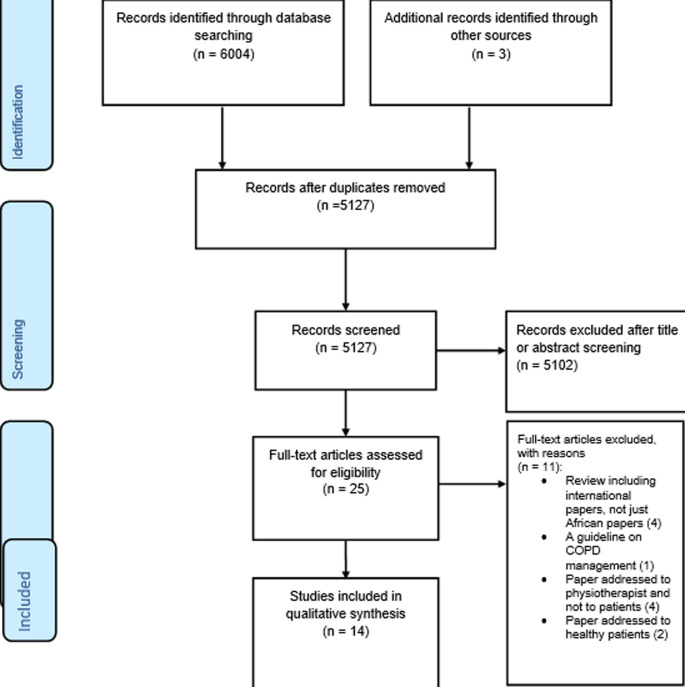
study flow chart

**Characteristics of included studies:** as shown in the PRISMA flow diagram (Figure 1), the initial searches provided 5127 non-duplicate records, of which 25 full texts were further assessed for eligibility and 14 studies were retained for qualitative synthesis. Eleven full texts were excluded because: Review including international papers, not just African papers (4), a guideline on COPD management (1), paper addressed to physiotherapist and not to patients (4), and paper addressed to healthy patients (2). Five thousand one hundred and two (5102) others do not rich the criteria (papers did not reported on pulmonary rehabilitation but on pulmonary diseases or others treatment and on other kinds of rehabilitation (neurological, orthopaedic). Geographically, included studies provided data from nine countries of which, four were from South Africa [12-15], two from Uganda [16,17], two from Nigeria [18,19] two from Zimbabwe [20,21], and one from Benin [22], one from Malawi [23], one from Egypt [24] and one from Democratic Republic of Congo (DRC) [25]. The study population was made of patients diagnosed for asthma, COPD, tuberculosis (TB) and post-tuberculosis lung disease (p-TBLD). Patients were also recruited from surgery department (who underwent a laparotomy), intensive care units (ICU) and from the human immunodeficiency virus/acquired immunodeficiency syndrome (HIV/AIDS) clinics. Table 1 provides a summary of observational studies assessing PR in Africa.

**Table 1:** summary of observational studies assessing pulmonary rehabilitation in Africa

Study ID, Reference, Country	Population	Aims	Findings
Morrow B *et al* 2016 [13], South Africa	18 COPD patients; 59.0±7.9yrs; <80% predicted FEV1	Effect of positioning and diaphragmatic breathing on respiratory muscle activity in a convenience sample of people with COPD, using sEMG	A Single diaphragmatic breathing session temporarily improved diaphragmatic muscle activity, without associated reduction in dyspnoea
Clarke H and M. Voss, 2016 [14], South Africa	12 COPD patients	If community-based, multidisciplinary team (home-based caregivers and supervised students) could improve functional status and quality of life of patients living with COPD in peri-urban setting	Home-based management of COPD patients by a multidisciplinary student team improves QoL and functional status of this patients in a low-income setting
Jones R *et al*., 2017 [16], Uganda	29 patients with p-TBLD; mean age 45 yrs	Assess a culturally appropriate PR program in Uganda for people with p-TBLD	PR for p-TBLD patients can be realize in Uganda
Jones R *et al*, 2018 [17], Uganda	42 patients with p-TBLD or COPD	Evaluate the lived experience of people with CRD, including physical and psychosocial impacts, and how this are addressed by PR	PR improve the physical, mental, and social functioning of CRD patients
Tadyanemhandu C and S. Manie, 2015 [20], Zimbabwe	137 ICUs patients (mean age = 36.0 yrs (SD = 16.6))	Describe the profile of patients and the current patterns of physiotherapy services delivered for patients admitted in five public hospital ICUs	Young patients Physiotherapy to prevent and treat respiratory complication
Tadyanemhandu C *et al*., 2018 [21], Zimbabwe	92 HIV/AIDS patients (mean age 41.3 (SD = 9.1) yrs)	Assess pulmonary conditions leading to hospital admissions in people living with HIV/AIDS at two central hospitals and the PR intervention received	Respiratory complication is one of the main causes of morbidity associated with HIV but PR is not offered to these patients
Kpadonou GT *et al*., 2011 [22], Benin	71 patients whounderwentlaparotomy	Effect of chest physiotherapy in patients undergoing laparotomy	Quicker improvement in respiratory and abdominal functions in most patients

sEMG: surface electromyography; HRQoL: Health-Related Quality of Life; QoL: quality of life IQR: interquartile range; FVC: forced vital capacity; FEV1: forced expiratory volume in 1 second, PEF: peak expiratory flow; ICUs: intensive care units; HIV/AIDS: human immunodeficiency virus/acquired immunodeficiency syndrome; COPD: chronic obstructive pulmonary disease, p-TBLD: post-tuberculosis lung disease, PTB: pulmonary tuberculosis, CRD: Chronic Respiratory Disease; PR: pulmonary rehabilitation, yrs: years; IG: intervention group; CG: control group

**Figure 2:**
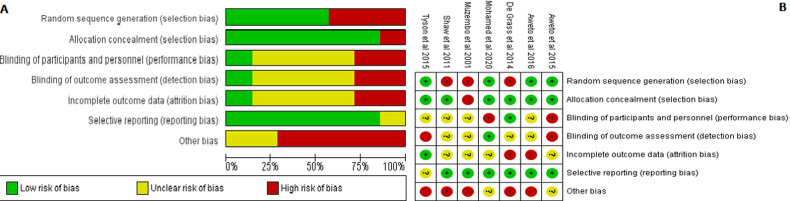
(A,B) quality of randomized controlled studies using the Cochrane risk of bias tool

**Risk of bias**: Figure 2 and Annex 1 summarize the authors´ judgment of risk of bias among included individual randomized studies using the Cochrane risk of bias tool and observational studies using the Newcastle Ottawa scale, respectively. The overall methodological quality assessment for interventional studies indicated a low risk of bias for selection and reporting bias while it showed an unclear to high risk of bias for performance, detection, and attrition biases. Further, among observational studies, the quality assessment of all the studies was high risk of bias with a substantial concern for lack of comparative groups.

**Effectiveness of pulmonary rehabilitation:** Table 2, Table 2 (suite), Table 2 (suite 1) provide an overview of the seven randomized controlled trials studies that reported effectiveness of the intervention, (two from South Africa [12,15], two from Nigeria [18,19], one from Malawi [23], one from Egypt [24] and one from Democratic Republic of the Congo (DRC) [25]). Studies differed regarding the type of intervention used. Differences were observed regarding the duration, frequency, and length of programs. In general, exercise programs lasted 10 to 30 minutes, conducted 2 or 3 times per week for 6 to 8 weeks and sometimes 16 weeks. Exercise capacity was assessed in three studies by the 6-minute walking test (6MWT) [15,24,25], and maximal power developed on cycle ergometer [25]. Significant improvements in exercise capacity were found [15,24,25]. Quality of life was measured in three studies [15,19,24], using the St Georges Respiratory Questionnaire (SGRQ) or COPD Assessment tool. There was no statistically significant difference between the intervention group and the control group in one study (p=0.789) [15], but significant improvements were observed in two others studies [19,24]. Most of studies reported significant improvement in lung function (FEV1, FVC, PEF, MVV, FEF, FEV1/FVC) [12,15,18,19,24,25] ((Table 2, Table 2 (suite), Table 2 (suite 1)).

**Table 2:** effectiveness of pulmonary rehabilitation in Africa

Reference, ID, Country	Design	Participants	Intervention	Outcome and key findings
B.S. Shaw and I. Shaw2011 [12], South Africa	RCT	88 asthmatics; aged 18-34 years; 60-80% of predicted FVC, FEV1 and/or PEF	8 weeks, 3 times/week of aerobic exercise (AE), diaphragmatic inspiratory resistive breathing (DR), and aerobic exercise combined with diaphragmatic inspiratory resistive breathing (CE)	FVC, FEV1, PEF, IVC, MVV: Significant improvement ofchest dimensions and kinematics (p ≤ 0.05) in AE, DR, and CE. All interventions significantly improved FVC, FEV1, PEF, and IVC. MVV improved following AE and CE [CE proved superior to AE at improving FVC (p = 0.001), FEV1 (p = 0.001), and IVC (p = 0.009)]. No significant change (p>0.05) in any of the measured parameters in the non-exercise CG Aerobic exercise +diaphragmatic inspiratory resistive breathing useful as an adjunct therapy for asthmatic patients
De Grass *et al* 2014 [15], South Africa	RCT	67 participants with PTB	6-week home- based PR program (low-impact exercises, wall push-ups, repeated sit to stand movements, walking)	FVC, FEV1, 6MWD: Significant difference between CG and IG for FVC (p=0.004; 95% CI: -0.36 to -0.07) and FEV1 (p=0.001; 95% CI: -0.33 to -0.08). Significant difference in distance covered between participants in the IG and CG (p=0.007; 95% CI: 15.37 to 92.7m) Motivation for the consideration and implementation of a PR program for PTB

**Table 2(suite):** effectiveness of pulmonary rehabilitation in Africa

Reference, ID, Country	Design	Participants	Intervention	Outcome and key findings
H. A. Aweto *et al*., 2015 [18], Nigeria	RCT	45 asthmatics patients; age ranged 18-48yrs	Aerobic or resisted exercise 10-15 minutes, 2 time per week for 6 weeks	SBP, DBP, HR, RPP, RR, FEV1, FVC, FEV1% Significant improvements for resisted exercise and counselling group in SBP: p=0.01, DBP: p=0.03, HR: p=0.02 and RPP: p=0.01 Significant improvements for aerobic exercise and counselling group RR: p=0.01, FEV1: p=0.01, FVC: p=0.01 and FEV1 %: p=0.02 Aerobic exercise more effective in improving pulmonary parameters; resisted exercise more effective in improving cardiovascular parameters
H. A. Aweto *et al*, 2016 [19], Nigeria	RCT	40 HIV patients (aged +18 yrs)	Aerobic exercise using bicycle ergometer for 30 min, 3 days per week for 6 weeks and counselling sessions for 30 min, once in 2 weeks	FEV1, FVC, PEF, respiratory symptoms, depressive symptoms, QoL: Significant differences between IG and CG mean (SD) changes in FEV1(P=0.001), FVC (p=0.001), PEF (p=0.001), respiratory symptoms (P=0.001) and depressive symptoms (P=0.001); significant improvement of QoL in IG (SGRQ: P=0.001) but no significant improvement in the CG Significant improvement of pulmonary functions and reducing of respiratory and depressive symptoms
J. MuzemboNdundu *et al*., 2001 [25], D. R. Congo	RCT	38 COPD/Asthma patients; 52±14 years; FEV1 1,37±0,62 (50 %pred)	PR program of 8 weeks, 3 times per week, with bronchodilator by aerosol, bronchial toilet, costo-diaphragmatic ventilatory exercises and exercise training	FEV1, 6MWD, maximal power developed on cycle ergometer: After rehabilitation program FEV1 increased from 1.37±0.62 (50% expected) to 1.54±0.69 (56% expected) (p<0.01); 6MWD (from 644±459m to 1213±569m, p<0.001) and Maximal power developed on cycle ergometer (from 45±20w to 73±37w, p<0.001) Improvement of quality of life of patients

**Table 2(suite 1):** effectiveness of pulmonary rehabilitation in Africa

Reference, ID, Country	Design	Participants	Intervention	Outcome and key findings
A. F. Tyson et al, 2015 [23], Malawi	RCT	150 adult patients who underwent exploratory laparotomy; median age: IG=35 yrs(IQR, 28-53 yrs) and CG 33 yrs (IQR, 23-46 yrs)	Post-operative deep breathing exercises and incentive spirometry	FVC No significant changes between IG and CG Addition of incentive spirometry to the treatment not recommended
Mohamed Sha**makh *et al***, 2020 [24], Egypt	RCT	60 COPD patients; Aged 40-65; (FEV1/FVC < 70% of predicted & FEV1 < 80% of predicted)	ACBT, PEP, acapella	FEV1, FEV1/FVC, 6MWT, QoL FEV1 improved in all groups; A: 2.64%, B: 8.92%, and C: 10.49%; Groups B and C had a significant difference from Group A (P=0.001 and P=0.008); FEV1/FVC improved in all groups; A: 3.71%, B: 7.73%, and C: 8.52%; Groups B and C had a significant difference from group A (P=0.024 and P=0.001); Walking distance in the 6MWT increased in all groups; A: 3%, B: 12.95%, and C: 20.09%; Groups B and C had a significant difference from group A (P=0.004 and P=0.013); QoL improved in all groups (all groups' test scores decreased) A: 4.36%, B: 22.85%, and C: 22.99%; Groups B and C had a significant difference from group A (P=0.001 and P=0.001). No significant difference between groups B and C for all value PEP and acapella improved moderate COPD pulmonary functions. ACBT alone showed improvements but to a lesser extent than Acapella and PEP

RCT: randomized controlled trial, QE: quasi-experimental study, P-PIC: pre- and post-interventional cohort study, AerG: Aerobic Group, ResG: Resistance Group, ConG: Concurrent Group, FVC: forced vital capacity, FEV1: forced expiratory volume in 1 second, PEF: peak expiratory flow, PIF: peak inspiratory flow, IVC: inspiratory vital capacity, FEF-25: forced expiratory flow at 25%, VO2 max: maximal oxygen uptake, MVV: maximal voluntary ventilatory, SBP : systolic blood pressure, DBP : diastolic blood pressure, HR : heart rate, RPP : rate product pressure, RR : respiratory rate, 6MWT: 6 minute walking test, CCQ: clinical COPD questionnaire score, BODE: body mass index, airflow obstruction, dyspnoea and exercise capacity, ISWT: incremental shuttle walking test, CRD: chronic respiratory disease, COPD: chronic obstructive pulmonary disease, p-TBLD: post-tuberculosis lung disease, PTB: pulmonary tuberculosis, PR: pulmonary rehabilitation; yrs: years; IG: intervention group; CG: control group; BP: blood pressure; PEP: positive expiratory pressure; ACBT: Active cycle of breathing technique; SD: standard deviation; QoL: Quality of Life.

**Pulmonary rehabilitation components:** Table 3 shows the components of PR programs. Exercise training (aerobic exercise) was included in five studies (walking, cycling and others aerobic exercises such as rower, step, whole-body exercise, climbing stairs and stretching) [12,15,18,19,25]. The most used component was breathing exercises reported in six studies [12,15,18,23-25]. Other components were education reported in three studies [15,18,19], home-exercises (in two studies) [12,15], resistance/strength training (in one study) [18], inspiratory muscle training (in one study) [12] and self-management (in one study) [12].

**Table 3:** components of pulmonary rehabilitation program in included effectiveness studies

	B.S. Shaw and I. Shaw 2011 [12]	De Grass *et al*. 2014 [15]	H. A. Aweto *et al*., 2015 [18]	H. A. Aweto *et al*., 2016 [19]	Muzembo Ndundu J *et al*. 2001 [25]	Tyson AF *et al*., 2015 [23]	Mohamed Shamakh *et al*., 2020 [24]	Total
Aerobic exercise – walking	✓	✓	✓		✓			4
Aerobic exercise–cycling				✓	✓			2
Aerobic exercise-other	✓	✓			✓			3
Resistance/strength training			✓					1
Breathing exercises	✓	✓	✓		✓	✓	✓	6
Inspiratory muscle training	✓							1
Training in activities of daily living								
Self-management	✓							1
Education		✓	✓	✓				3
Energy conservation								
Nutritional support								
Smoking cessation								
Psychosocial support								

### Survey

**General consideration:** E-mail addresses were hard to find and sometimes when they were found, they did not even exist anymore. Some correspondents did not answer directly to our e-mail, and some never responded. Of 39 surveys mailed to health professionals around Africa, only 14 (35,8%) were returned (from South Africa, DRC, Uganda, Nigeria, Angola, Cameroon, Niger, Senegal) from which seven health professionals (physiotherapist, pulmonologist, and physiatrist) reported having at least one or more PR programs in their facilities such as inpatient, outpatient, maintenance and/or home-based programs. One respondent (from South Africa) reported online support via an email/Skype program. The remaining seven respondents from South Africa, Senegal, Niger, Angola, Nigeria, and DRC reported the absence of a program in their facilities. The others 25 health professionals never answer to the mail.

**Program characteristic, components, follow-up, and health worker:** in- and outpatient programs were the most reported programs with a duration of 60 minutes (for each session) in general for three (outpatient program) or six (inpatient program) days per week with a length of six (inpatient program) or eight (outpatient program) weeks per session (Annex 2). Aerobic exercises, mostly walking (100% of in- and outpatient programs) and strength training of upper and lower extremities (100% of in- and outpatient programs) were the most used exercise modalities in PR programs, followed by breathing exercises (100% of in- and outpatient programs) (Annex 2). Home exercise prescription was provided for outpatient (86%) and for inpatient (80%) programs. There was little use of education (20% of inpatient, 57% of outpatient programs) and psychological support (20% of inpatient and 43% of outpatient programs) reported in PR programs. The distribution of follow-up components is summarized in Annex 2. Follow-up was added as part of the pulmonary rehabilitation program in all facilities that reported having a PR program in this study. Reassessment was the most used follow-up component (80% of inpatient and 100% of outpatient programs) and supervised exercises the least (0% of inpatient and 28% of outpatient programs). Pulmonologist, physiatrist, internist, and physical therapist were the most represented health professionals (100%, 71%, 57% and 43% respectively of outpatient program), while social worker (14% of outpatient program) and psychologist (28% of outpatient) were the least represented (Annex 2). Nurse and spiritual leader were absent in the reported programs. Other healthcare professionals, i.e., resuscitator/anaesthetist, thoracic surgeon, paediatrician was part of the PR program team in the Kinshasa facilities.

**Outcome measures:** outcome measures are shown in (Annex 3). The 6-minute walking test was the most frequently used measure (100% of in- and outpatient programs) followed by St Georges Respiratory Questionnaire (100% of in- and outpatient programs) and simple spirometry with bronchodilator response (100% of in- and outpatient programs). The Chronic Respiratory Disease Questionnaire, constant work rate test, 12-minute walking test and shuttle walk test were not used.

The interest of this review and survey was to investigate the current evidence and practice of PR in Africa (North and Sub-Saharan Africa). In the systematic review, 14 studies were included and showed a disparate existence of structured PR in some African facilities indicated for COPD patients, asthma, pulmonary tuberculosis and post-tuberculosis lung disease patients, surgical patients, ICU patients and HIV/AIDS patients. The improvement of quality of life of CRDs patients was also shown.

A web-based survey yielded a very limited response rate despite several reminders and thus reduces the generalizability of our survey´ findings. Additionally, due to the significant level of heterogeneity, we were unable to do meta-analyses. The interpretation of the results must therefore be made with caution.

Nevertheless, from the respondents, we noted that despite the high burden of CRDs in the continent [26], PR as a structured practice is still not well established as a popular treatment for CRDs in Africa. We found that outpatient (100%) PR program was offered by the respondents in the survey study with a median of six patients per program, attending for three days a week, for sixty minutes (one hour) per day over eight weeks. PR program offered to inpatient was shorter than outpatient program (six weeks) but more intense, four to seven days a week for sixty minutes. Although some of these findings might compare well with the international guidelines´ requirements, considering the paucity of data and the scarcity of equipment, we will recommend that throughout the development of new PR in Africa, instead of constructing de novo guidelines- or simple adopting other guidelines, combining local experiences, local evidence to adapt current international guidelines might support faster implementation of PR practice in the African region.

The systematic review found that programs take 10 to 30 minutes, 2 or 3 time per week for 6 to 8 weeks. The British Thoracic Society guideline on PR also recommended PR program 2 to 3 time per week for 6 to 12 weeks and is in line with current literature [27]. Moreover, endurance training was largely used with walking (100% of program in the survey) and in the review (four studies [12,15,18,25], out of seven reported in effectiveness studies) as it is suggested by the American thoracic society/European respiratory society statement (ATS/ERS) statement [6] and other professional societies [28]. Walking is easier to practice and cheap since it does not necessarily require sophisticated equipment (such bicycle ergometer, treadmill). In addition, walking is very important as daily life activity.

Furthermore, breathing exercise was among the most used component for PR practice in Africa and this was also reported in both inpatient and outpatient program in our survey. This type of exercise aims at modifying respiratory muscle recruitment and reduces dyspnoea in COPD patients [29]. However, its effect on dyspnoea, exercise capacity and wellbeing it is unclear despite the fact that some studies have reported positive short-term physiological effects of breathing exercises in COPD patients [29].

Unfortunately, despite its well-known importance as PR component [6] and evidence of effectiveness in COPD self-management skills [30], patient education was only used in 3 studies in this paper [15,18,19].

Frequently measured outcomes included 6MWT (100% for inpatient, 86% for outpatient of the survey and reported on three papers in the review [15,24,25]) which is the most established field walking test and is more used because it is low-cost, necessitates little equipment, it is suitable for evaluation in the community setting, and it is considered to be more reflective of daily living than laboratory-based treadmill or cycle ergometer tests [6]. The 6 MWT provides data on the patient's physical activity level which can be a better indicator of the patient's ability to perform daily activities [31]. Improvement in walking distance was effective and common as reported in several included studies across Africa for COPD [24,25], asthma [25] and post-tuberculosis [15] patients after PR according to ATS/ERS statement [6].

Lung function measurement was mostly evaluated with simple spirometry with bronchodilator response in inpatient (100%) and outpatient (86%) programs in the survey study. Though some studies reported statistically and clinically significant improvements in Forced Vital Capacity (FVC), Forced Expiratory Volume in 1 second (FEV1), FEV1/FVC ratio in patients with asthma, pulmonary tuberculosis and COPD in the review, it is known that a short-term improvement of lung function in COPD patients after a PR program is unexpected [32]. In accordance with the current opinion, reversing the progressive decline in lung function in COPD patients is unlikely [33].

All pulmonary rehabilitation programs have the same essential characteristics, but the resources available, the program setting, structure, staff and duration vary considerably from one health system to another [6]. Currently, clear evidence to assign the right patient, in the most necessary setting, for the necessary rehabilitative treatment, including medical and non-medical therapies tailored to the patient is lacking, there is also no consensus of experts around the world, especially because of all the differences in local situations [34]. It is therefore evident that PR program in Africa have to be adapted to the patients culture, habits and systems to make it more accessible to Africans.

Consequently, further studies on PR are warranted to accelerate the adaptation of guidelines in Africa while considering health system constraints especially during COVID-19 era. PR programs delivery was severely impacted by the coronavirus disease 19 (COVID-19) pandemic what prompted to review the methods of application of exercises [35]. As tele-rehabilitation becomes an interesting tool for PR in developed countries [35], in Africa it becomes crucial to find a program that would keep patients and caregivers safe. An inexpensive program would be ideal for Africans like dance which doesn´t require expensive device. Dance can be practiced with physical distance and even outdoor and could improve physical performance, function, mood and social engagement, and is a core aspect of societies and cultures globally [36]. It would be a good program for futures researches in the area of PR in Africa if combined to adapted patients educations practices.

## Conclusion

Despite the relative presence of pulmonary rehabilitation in Africa, its practice is still limited across many countries with high disparities within countries. Considering, the high burden of chronic respiratory diseases in Africa, there is a necessity to conduct robust studies testing local practices (such as traditional dance) to help engineering adapted PR guidelines for easy implementation in poor resource settings where more chronic respiratory diseases are reported.

### What is known about this topic

- Pulmonary rehabilitation increases the quality of life of patients with chronic respiratory disease;
- Pulmonary rehabilitation works in occidental settings but given the difference in resources and trained personnel in Africa, it is important to understand the existing evidence for pulmonary rehabilitation in the continent.

### What this study adds

- Pulmonary rehabilitation is underutilized in North and Sub-Saharan Africa;
- Pulmonary rehabilitation program varies in African countries but generally respects guideline of occidental societies which do not have same realities with African countries in general.

## Annexes

**Annex 1**: quality of observational studies Assessing Pulmonary Rehabilitation in Africa using the Newcastle Ottawa scale

**Annex 2**: program characteristics, components, follow-up, and category of health care workers involved in pulmonary rehabilitation in Africa

**Annex 3**: reported outcome measurements at the start and the End of the Program
